# New Plastitar Record for the Mediterranean Sea: Characterization of Plastics and Tar from the Salento Peninsula (Ionian Sea)

**DOI:** 10.3390/toxics13010013

**Published:** 2024-12-26

**Authors:** Silvia Fraissinet, Emanuele Mancini, Chiara Funiati, Caterina Martino, Giuseppe Egidio De Benedetto, Chiara Roberta Girelli, Francesco Paolo Fanizzi, Genuario Belmonte, Stefano Piraino

**Affiliations:** 1 Department of Biological and Environmental Science and Technology (Di.S.Te.B.A.), University of Salento, Campus Ecotekne, Via Prov. Lecce-Monteroni, 73100 Lecce, Italy; silvia.fraissinet@unisalento.it (S.F.); chiara.girelli@unisalento.it (C.R.G.); fp.fanizzi@unisalento.it (F.P.F.); genuario.belmonte@unisalento.it (G.B.); stefano.piraino@unisalento.it (S.P.); 2 National Biodiversity Future Center (NBFC), 90100 Palermo, Italy; 3 Academy of Fine Arts, Via Libertini, 3, 73100 Lecce, Italy; funiatichiara@gmail.com; 4 Department of Earth and Sea Sciences (DiSTeM), University of Palermo, 90100 Palermo, Italy; caterina.martino@unipa.it; 5 Laboratory of Analytical and Isotopic Mass Spectrometry, Department of Cultural Heritage, University of Salento, 73100 Lecce, Italy; giovanni.debenedetto@unisalento.it; 6 CoNISMa Consorzio Nazionale Interuniversitario per le Scienze del Mare, 00196 Rome, Italy

**Keywords:** Italian coats, marine pollution, tar, polyethylene, oil, microplastics

## Abstract

The various forms of anthropogenic pollution are regarded as a serious threat to marine coastal areas. The overproduction and mismanagement of petroleum derivatives, such as tar and plastics, have resulted in a significant correlation between these two pollutants. The aggregation of tar, microplastics (MPs), and natural materials can create plastitar blocks, which are common in coastal areas. These raise concern about the undeniable negative impact on the marine ecosystem and the associated biota, and serve as a recognizable and understandable indication of environmental decline. Here, the composition of the 11 plastitar blocks collected on the Ionian side of the Apulia region (Italy) was characterized both in tar and plastics using nuclear magnetic resonance (NMR) spectroscopy and Fourier transform infrared (FTIR) spectroscopy, respectively. Of the 250 particles extracted from the tar, 208 were identified as plastics, predominantly Polyethylene. The majority of these were in the form of pellets (90%), with fragments accounting for 5% and films and filaments representing the remaining 5%. This study provides new data that can be used to enhance the understanding of the distribution and baseline information about this novel form of pollution in Italian waters.

## 1. Introduction

The presence of plastic in every compartment of the globe is not just a problem; it is a pressing global crisis [1,2,3]. With plastic production worldwide continuously and dangerously increasing year after year, reaching 400.3 Mtonnes in 2022 [4], and a mere 9% of this plastic being recycled [5], the enormity of the problem is starkly highlighted. The consumption of single-use items, accounting for approximately 50% of global plastic production, is still a major concern [6,7,8]. Plastic can reach the marine environment from a variety of sources, including the fishing and aquaculture industries (e.g., rope, waste, fishing gear, and nets), ships and vessels used for commercial and transportation purposes, and also land-based origins like mismanaged waste or urban litter [9,10,11,12,13]. Plastic litter can be classified according to size: macroplastics (>2.5 cm), mesoplastics (from 5 mm to 2.5 cm), and, most infamous, microplastics (MPs, <5 mm) [14,15]. The latter can be derived from the degradation of larger plastic items (macro- and mesoplastics) following UV radiation exposure and mechanical embrittlement (secondary microplastics), or they can be intentionally manufactured for human purposes (primary microplastics). They have been detected in water, sediments, and, of course, various species of marine animals [16,17,18,19].

Furthermore, oil also represents one of the most historically concerning pollutants in the marine environment. The routine or accidental release of drilling activities, transport, and waste mismanagement into the sea can have significant environmental consequences, particularly for marine fauna [20,21,22,23]. The coastline is one of the most exposed areas where oil can persist for long periods of time due to the combined effect of currents, winds, and tides which can enhance its permanence in the area [24,25,26,27].

As a marker of the Anthropocene, a new material named plastitar, which combines oil derivatives (tar) with plastics (mainly primary origin), has been recently described as “an agglomerate of tar and plastics (mainly microplastics), which are amalgamated and attached to a rock surface” [28]. The authors put forth a hypothesis regarding the genesis of this material, proposing that interactions between tar and floating plastics are responsible for its formation. Due to the fluctuations in temperature and UV light throughout the diurnal cycle, tar undergoes a cyclical process of hardening and softening. During this process, floating plastics on the sea surface may become entrained and mixed within tar structure. Ultimately, the volatile components evaporate, and the high molecular weight fraction hardens, leading to the solidification of the blocks. These blocks incorporate not only plastics, but also natural materials [28]. An alternative hypothesis for the formation of plastitar is that the hardening and weathering process occurs while the tar is already attached to the rock. Consequently, when plastics or other materials come into contact with the surface, they can become entrapped in the soft crude oil mixture and remain embedded within it during the hardening phase [29].

Ellrich and coworkers [30] conducted a thorough investigation into this phenomenon, uncovering that reports of plastic embedded in tar have been documented since 1973. Furthermore, over the past five decades, ten studies have been conducted on this novel material, with eight of these plastitar records occurring in proximity to the oil transportation routes from the North Atlantic to the Sea of Japan [30]. A limited number of studies have been conducted on plastitar in the Mediterranean Sea. The initial observation was documented in 2011 along the Maltese coastline, where concentrations reaching several thousand pellets per square meter were identified within tar deposits attached to emerging and intertidal rocks [31]. In 2020, Fajković et al. [32] published a report in which they described the presence of a new phenomenon on Croatian islands. They termed these objects “plasto-tarballs”, and they proposed that they function as a sinkhole for MPs due to their high prevalence in the tar matrix [32]. A more recent study has provided detailed qualitative and quantitative data on the occurrence of tar and microplastics (MPs) in four distinct regions of the Mediterranean Sea: The North Mediterranean, the Southern Adriatic, the Southwest Mediterranean, and the Western Mediterranean [33]. The authors observed an exceptionally high concentration of pellets within their tar samples, indicating a pervasive distribution of plastitar across the investigated sites, which have previously been identified as hotspots for both MPs and oil pollution [24,32,34].

Despite general concern and the enactment of legislation to protect the environment from accidental spills, the International Convention for the Prevention of Pollution from Ships [35], global oil consumption and marine transport activities are nowadays increasing, similar to plastic production [29], underscoring the necessity for the dissemination of updated information regarding the interaction between these two forms of pollution. Considering the preceding publications, numerous areas of the Mediterranean Sea, particularly the Italian peninsula, have been subjected to investigation, including the Apulian region. To date, the Northern Ionian Sea, extending between Punta Alice (KR) and Santa Maria di Leuca (LE), has yet to be investigated [36]. Furthermore, this area is home to the Gulf of Taranto, which is characterized by the presence of intense commercial and cruise ship traffic. The present study investigated eleven plastitar blocks collected along the Ionian Apulian coastline. The objectives of this study were threefold: firstly, the extraction of the plastic particles from the matrix and their qualitative and quantitative characterization; secondly, the identification of the tar composition; and thirdly, the study of the plastic concentration in the analyzed tar blocks. This study will contribute further data to increase the baseline of information related to this new trend in marine pollution, which combines two of the most serious environmental problems of the 20th century: plastic pollution and oil spills.

## 2. Materials and Methods

### 2.1. Collection and Storage of Plastitar Block

**Figure 1:**
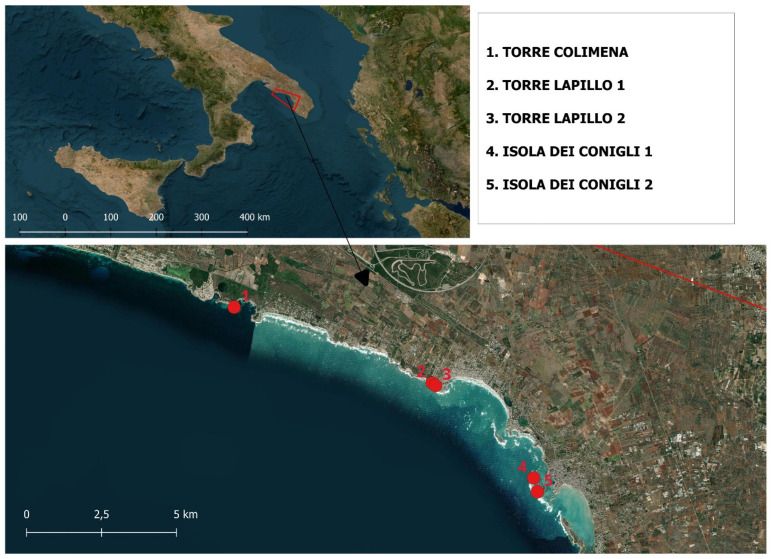
Map of the sampling area. Red markers identify the sampling points.

Eleven plastitar blocks were collected (Figure S1 in Supplementary Materials) from five different sites located along the Ionian coast of the Apulian Region, in the summer season (July 2024) (Figure 1; Table 1). Each block was gently removed from the rocks using a chisel as leverage. Once in the laboratory, each plastitar block was cataloged, wrapped in white paper, and separately stored in a cool and dry place, away from sunlight to preserve its integrity, avoiding possible melting due to the high summer temperature.

### 2.2. Plastic Extraction

Plastitar blocks were photographed and weighed before extracting plastic from the tar structure. Each plastitar block was broken manually, taking advantage of its malleability, and carefully explored. Plastic extraction was performed using stainless-steel tweezers and the particles were carefully placed in 10 mL glass vials. Then, 5 mL of hexane 96% (Carlo Erba, Milan, Italy) was added to each vial in an ultrasonic bath (Branson 3510, Sigma Aldrich, Milan, Italy) (15 min treatment) to remove tar residues that can interfered with subsequent spectroscopic analyses. This cleaning treatment was repeated multiple times (at least six), substituting the dirty hexane full of tar residues with a clean amount of reagent each time.

### 2.3. Plastic Morphological Characterization

The particles extracted from each sample were collectively weighed using an analytical balance (NAPCO JA-210) and singularly photographed using Leica Stereoscope equipped with Nikon Imaging Systems (NIS) Elements software (version 4.60) to report the shapes and colors. The size of plastics was instead determined using ImageJ software [37].

### 2.4. Plastics Spectroscopic Characterization

The Agilent Technologies FTIR spectrometer model Cary 680 equipped with a DTGS detector and Pike Miracle ZnSeGermanium ATR attachment (Agilent Technologies, Milan, Italy) was used to spectroscopically analyze the particles separated from tar. The measures were carried out in ATR-FTIR mode in the spectral region of 600–4000 cm^–1^, where the resolution was 4 cm^–1^ and the number of scans was 32 for each spectrum. The FTIR spectra were acquired using ResolutionsPro (Version 5.20 CD 84) software and imaged using Origin Pro 2016 (64 bit). All the particles present in the samples were analyzed; 40% of each particle morphology and color underwent ATR-FTIR characterization just in the case of P4, P7, and P11, due to the high number of particles found.

### 2.5. Tar NMR Sample Preparation and Characterization

Sample preparation for NMR analysis was performed by dissolving 10 mg of plastitar compound in 600 µL of deuterated chloroform (CDCl_3_) containing 0.03 *v*/*v*% TMS (sodium salt of trimethylsilyl propionic acid) as a chemical shift reference. After filtration, the resulting solution was transferred to a 5 mm NMR tube. Three technical triplicates were performed. For fraction proton distribution, an NMR sample was prepared by dissolving the same amount of compound in 500 µL dichloromethane (CD_2_Cl_2_). Spectrum acquisition was performed at 300 K on the Bruker Avance III 600 Ascend NMR spectrometer (Bruker, Ettlingen, Germany), operating at 600.13 MHz for ^1^H observation and equipped with a TCI cryoprobe incorporating a z-axis gradient coil and automatic tuning matching (ATM). A one-dimensional experiment (zg Bruker pulse program) was run with 256 scans, 64 K time domain, spectral width of 20.0276 ppm (12,019.230 Hz), acquisition time of 2.73 s, relaxation delay of 2 s, and pulse p1 of 8.6 s. The resulting FIDs were multiplied by an exponential weighting function corresponding to a line broadening of 0.3 Hz before Fourier transformation, automated phasing, and baseline correction. The resonance identifications were based on ^1^H and ^13^C assignment by 1D and 2D experiments (^1^H-^13^C hsqcetgpsisp2.3, and hmbcgplpndqf and 1H decoupled 13C, hr_zg0dc Bruker pulse program), and by comparison with the literature data [38,39].

### 2.6. Contamination Control

During both the field and laboratory procedures, measures were implemented to minimize contamination. While conducting field activities, a metal chisel was used to remove plastitar blocks from the rocks, and samples were preserved by covering them with paper. In the laboratory, all staff wore cotton gowns and/or cotton attire, nitrile gloves were utilized throughout all laboratory processes. Every procedure was carried out in glass containers using metal tools and tweezers. Each container and tool was cleaned using MilliQ water filtered through a 0.1 µm-pore-size PES filter (Steriltech, 47 mm 0.1 μm pore size) and paper.

## 3. Results

### 3.1. Tar NMR Characterization

**Figure 2:**
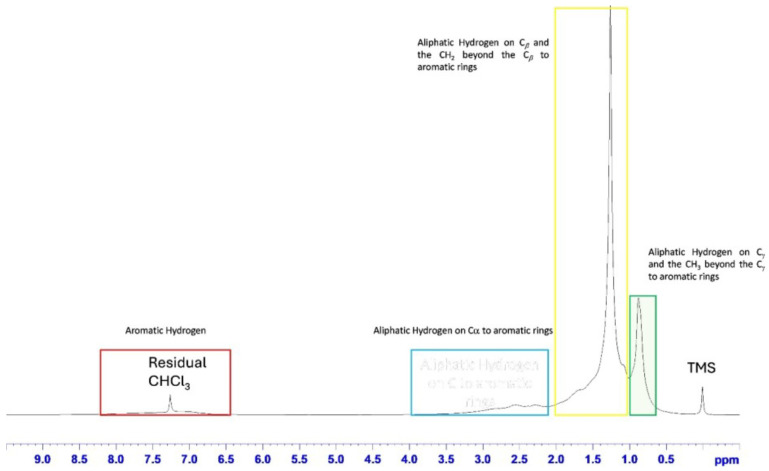
^1^H-NMR spectrum of the plastitar compound in CDCl_3_. Diagnostic proton assignments are indicated.

The structural assignments of plastitar compound ^1^H NMR spectrum (Figure 2) showed the presence of four main spectral regions, as previously described. Intense signals at 0.88 ppm (carbon at −15–24 ppm) and 1.26 ppm (carbon at 23–40 ppm) were ascribed to aliphatic hydrogens on C_g_, the CH_3_ beyond the C_g_ to aromatic rings and on C*_b_*, the CH_2_ beyond the C*_b_* to aromatic rings, respectively. Two broad signals were identified: in the range of 2.00–4.00 ppm (carbon at 14–46 ppm), resonances of aliphatic hydrogen on C*_a_* to aromatic rings; and in the range of 6.5–8.00 ppm, aromatic hydrogen signals (carbon at 120–130 ppm).

The assigned spectral regions were then integrated after normalization, with the aim of providing the fractional proton distributions. In order to remove the chloroform signal in the aromatic region, the integration procedure was performed on a dichloromethane solvent NMR spectrum, with a residual solvent signal at 5.3 ppm (Figure S2 in Supplementary Materials). The hydrogen distribution, resulting from the NMR spectrum integration, revealed the highest percentage (~67%) of aliphatic protons on C*_b_*, the CH_2_ beyond the C*_b_* to aromatic rings with respect to the other aliphatic protons (7.68 and 21.82 for hydrogen on C_a_ to aromatic rings and C_g_ and CH_3_ beyond the C_g_ to aromatic rings, respectively).

### 3.2. Plastic Morphological Characterization

From the eleven plastitar blocks, 250 particles were extracted; 42 were materials with a natural origin like seeds, rocks, and shells, while 208 were plastics, with a concentration of 2.42 plastic items/g of tar. Most of the particles found were microplastics (75%), 24% were mesoplastics (5 mm to 2.5 cm), and just 1% was macroplastics (>2.5 cm). Pellets represented 90% of the plastics found, 5% were fragments, and the rest were films and filaments. The most frequently found pellets were yellowish. The average weight of the tar blocks was 85.69 g, while the average weight of the plastics extracted from them was 0.52 g (Table 2). Some pellets were melted together, while others reported signs of environmental exposure with some calcification residues on their surfaces (Figures S3 and S4, respectively, in Supplementary Materials).

### 3.3. Plastic ATR-FTIR Characterization

**Figure 3:**
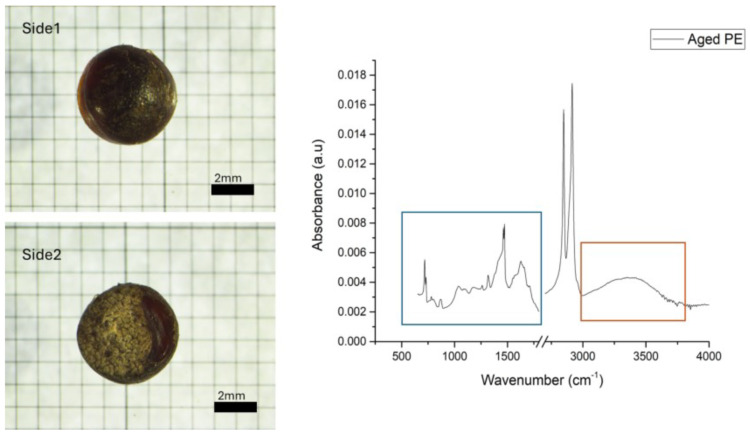
A small dark pellet was photographed on both sides and showed signs of damage: discoloration and embrittlement on Side 2 (scale bar 0.5 mm), and analyzed by ATR-FTIR spectroscopy. The resulting spectrum was identified as aged PE due to the presence of bands, highlighted in the blue and red boxes, referring to weathering processes.

A total of 104 plastics were analyzed; 89% of those particles was polyethylene (PE), 6% was polypropylene (PP), and 5% was cellulose. Some of the spectra presented irregularity in the fingerprinting region (500–1500 cm^−1^) and the region relative to OH stretching (3000 to 3600 cm^−1^), which were ascribed to the aging process due to environmental conditions (Figure 3).

**Figure 4:**
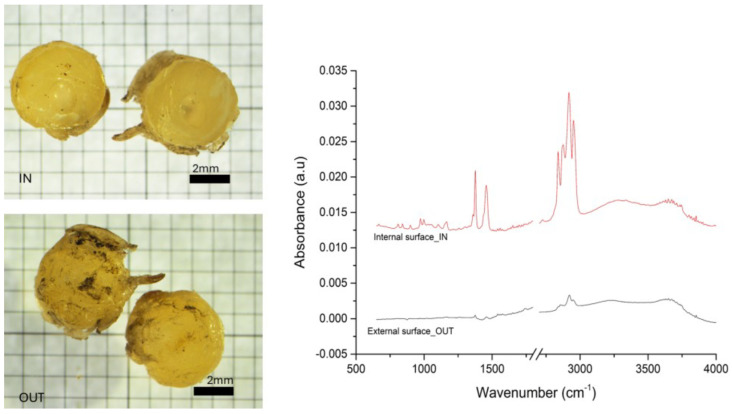
Yellowish pellet cut in half. Both halves were photographed, the outside surface is shown in the picture on top (OUT) and the inner part in the photo below (IN) (scale bar 0.5 mm). Both were analyzed by ATR-FTIR spectroscopy. The resulting spectra were identified as weathered PP (External surface_OUT) and PP less exposed to environmental aging (Internal surface_IN).

To better investigate the possible weathering that occurred on the recovered plastics, a pellet of which the outer surface was particularly damaged, which resulted in a spectrum difficult to identify, was cut in half to analyze the internal one, showing a defined spectrum of PP, and highlighting how the external parts of plastics can be highly weathered while the inner side is still well preserved (Figure 4). Also, in the PP spectrum, it is possible to see the big bands in the OH region (3000–3500 cm^−1^), while the bands in the fingerprinting region are very weak. This situation can indicate a high level of weathering, as can also be seen by the pictures comparing the outside and inside of the pellet.

## 4. Discussion

To achieve a comprehensive understanding of the tar matrix chemical composition of the plastitar samples, proton nuclear magnetic resonance (^1^H-NMR) analysis was performed. One of the key advantages of NMR is its ability to simultaneously examine multiple components of a mixture using a single ^1^H-NMR spectrum, allowing for the evaluation of the relative amounts of aliphatic and aromatic hydrogen in the mixture [40,41,42]. This technique is commonly employed for characterizing both synthetic and natural compounds. Unlike conventional analytical methods, NMR spectroscopy does not require the pre-treatment of samples, reducing the time and effort involved in sample preparation. Additionally, it is considered environmentally friendly due to the minimal use of solvents and reduced waste generation [43]. The matrix was identified as bitumen. As previously reported by Oliviero Rossi and coworkers [39], the resonances assigned to olefinic protons, in the expected range of 4.5–6.00 ppm, were not observed, as also confirmed by the assignments in the 2D ^1^H ^13^C hsqc spectrum (Figure S5 in Supplementary Materials), which means that the amount of olefinic hydrocarbons, although present, is negligible. On the other hand, the aromatic proton percentage showed the lowest value (3.64%), as already reported for industrial bitumen [39]. Bitumen possesses distinct chemo-mechanical characteristics, which is why it is currently one of the primary components in various industrial products. As a product derived from crude oil, it is classified as a viscoelastic material exhibiting adhesive and waterproofing features, and its chemical composition is largely influenced by its source (crude oil) and the refining process, both which contribute to its distinctive chemical and physical traits [39,44]. Chemically, bitumen is composed of roughly 80% carbon and 15% hydrogen, with the remainder made up of two categories of atoms: heteroatoms and metals [45].

The plastis found within the tar matrix were predominantly polyethylene (PE) and polypropylene (PP) in pellet form, followed by fragments and films. Microplastic was the most abundant size fraction, a finding that aligns with those of other authors in similar studies [28,31,33]. Globally, pellets represent the second most significant source of primary microplastics in the environment, with an estimated 450 tonnes of items spilled in oceans and seas worldwide annually [46]. Nevertheless, the existing literature frequently indicates that only 2.2% of plastic particles identified on Mediterranean surface waters is pellets [11]. It has been hypothesized by some authors that the high abundance of these particles in the tar blocks may be related to a particular association with oil during the formation of the plastitar [33]. Polyethylene and polypropylene were identified in significant quantities in this and other studies on plastitar. These are the most produced plastics worldwide [4] and the most common polymers found in marine water surfaces due to their low density [47]. This supports the theory that plastic and tar can interact on the water surface and, in the second stage, reach the shores, where they harden and adhere to rocks.

FT-IR spectroscopy is widely regarded as one of the most prevalent and suitable techniques employed in microplastic research [48]. Furthermore, this technique can be employed to highlight and measure the changes in the chemical bonds of plastic due to environmental aging [49]. The attenuated total reflection (ATR) configuration is a commonly employed approach, typically utilized for the analysis of larger particles [46]. In fact, at the interface of the crystal, where the internal reflection of the infrared beam occurs, a stationary evanescent wave is found and interacts with the sample: the limited sampling depth of the evanescent wave permits the analysis of high-absorbing substances, such as polymers, and it obviates the necessity for sample preparation, the only requirement being the intimate contact of the sample with the internal-reflection crystal. The resulting IR spectrum of the material pressed onto the surface is comparable to the one obtained by transmission after a simple elaboration of the spectrum (ATR correction).

As illustrated in Figure 1, the PE spectra display the emergence of new bands within the 1510-to-1770 cm^−1^ range of absorbance. These bands exhibit centered peaks at approximately 1614 cm^−1^ and a broad band at 3100–3600 cm^−1^, which is associated with the vibrational stretching of OH groups. These bands can be attributed to the oxidation of the PE surface during the aging process, as reported by other authors [49,50,51]. In particular, thermo-oxidative degradation can be more extensive in dark-colored plastics due to the potential for further temperature increases resulting from the accumulation of heat absorbed by the material from solar infrared radiation [52]. Regarding the PP spectrum of the external pellet surface, the characteristic bands of PP are almost undetectable, indicating a high level of degradation. In instances where plastics have been subjected to significant weathering, certain vibrational bands may not be discernible [53]. Furthermore, the prominent band in the OH stretching region is present in both spectra (internal and external surfaces), which may be attributed to prolonged exposure to water. The photodegradation of plastic debris typically commences with photo-oxidation, which initiates on the external surface that is exposed to environmental factors. Subsequently, due to the high UV-B radiation extinction coefficient in plastics and the presence of additives that can impede oxygen diffusion, the degradation can be localized outside [50,54], thereby preserving the core, as evidenced by our IR spectra. An alternative hypothesis for this deterioration is that the plastics were embedded in black tar material and exposed to solar radiation on the beach. This matrix contains chemicals that are prone to photo-oxidation [55], suggesting that the permanence in the black tar matrix may have accelerated the degradation pathway of the plastics. The surface may deteriorate in several ways, including the formation of pits, fine cracks, or a loss of color and strength. The color of the analyzed pellet provided further corroboration of these findings. The environmental weathering process resulted in significant damage to the surfaces and morphologies of the samples, as illustrated in Figures S2 and S3 of the Supplementary Materials. In particular, the white or yellowish hue was indicative of the loss of color that is consistent with prolonged persistence in the environment [47].

Despite the recent surge in interest surrounding novel plastic forms, the interaction between plastics and tar has been documented since the 1970s. However, the majority of published works on this topic have been conducted in the northern hemisphere. Frequently, plastic and tar have been observed along coastlines exposed to strong winds and intense offshore water currents [28,30,33]. This evidence supports the hypothesis that plastics and tar may accumulate in these circulation areas before being transported to the coastlines by wind-driven currents. This may account for the presence of this considerable quantity of polyolefin, which is typically composed of floating plastics within the tar matrix. However, Saliu and coworkers [33] also reported that they examined tar residues carried by the sea, and found no evidence of plastic. The formation and accumulation pathways of plastitar remain unclear, with potential variations at the regional scale. These pathways are likely linked to wind direction and the levels of pollution from plastics and oil [30].

The recently reported and defined new types of plastic waste represent a significant environmental risk, particularly in marine ecosystems. This is because they can break down and cause biomagnification, which, in turn, results in harmful effects on various organisms, as well as increased pollution levels that create unfavorable living conditions. The toxic substances present in tar, such as polycyclic aromatic hydrocarbons (PAHs), are harmful to marine organisms and can also accumulate in their bodies, leading to a range of adverse effects, including internal injuries and oxidative stress. Similarly, microplastics, due to the chemical additives and persistent substances that remain in their structure and their small size and shape, are responsible for direct and indirect adverse effects on marine fauna [28,56,57].

The presence of plastitar represents a significant threat to the marine ecosystem, with the potential for unknown environmental consequences. This highlights the urgent need for further data to better understand the extent of the problem and to conduct a thorough investigation into the sources and impacts of this phenomenon. Furthermore, there are still some unanswered questions regarding these emerging plastic litter variants. Consequently, further research is required to gain a full understanding of the potential implications regarding the origin and the presence of plastitar on shores worldwide. It is likely that the extent of data concerning this pollutant is more widespread than currently assumed. In this context, we present the first report on the presence of plastitar in the Salento region, thereby expanding our understanding of the distribution of this novel material along the Italian coastline.

## 5. Conclusions

In a world where plastic production and oil spills are dangerously increasing, plastitar formation could be accelerated or promoted, leading to a higher amount of this plastic variant being washed out to beaches and rocky shores. This is not a new phenomenon, and the little information available about it is concerning. Further research is needed to standardize the methods for sampling, the extraction of plastics from it, their cleaning, and characterization. Tar profiles and dataset, the same as plastic spectra, should be implemented and shared to deeply investigate the features of these materials after environmental exposure. Efforts are needed to expand knowledge about the possible direct and indirect impact of plastitar and other new plastic variants on marine coastal environments and communities. These new types of plastic waste can be red flags to underline how much the pollution problem needs to be managed at the source, which requires new ways to handle recycling systems and more efforts in controlling oil transportation routes. This needs to be managed at the international level, promoting regulations and guidelines on a global scale to face, what appears to be, a global impact.

## Figures and Tables

**Table 1:** Coordinates of the sampling points.

ID	Latitude	Longitude
1	40.29488	17.7553094
2	40.28286	17.8368281
3	40.28231	17.8383301
4	40.2602	17.8824595
5	40.25637	17.8848178

**Table 2:** The table summarizes the plastitar blocks and plastic weight. In the last column, the number of plastic items per block is shown.

ID	Tar Weight (g)	MPs Weight (g)	N of MPs
P1	74.7	0.257	15
P2	43.7	0.016	1
P3	29.6	0.047	2
P4	46.3	0.526	29
P5	88.8	0.183	3
P6	72.8	0.446	6
P7	131.9	1.207	65
P8	118.5	0.187	2
P9	82	0.089	2
P10	62.9	0.122	6
P11	191.4	2.64	77

## Data Availability

The original data presented in the study are included in the article/Supplementary Materials; further inquiries can be directed to the corresponding author.

## Supplementary Materials

The following supporting information can be downloaded at: https://www.mdpi.com/article/10.3390/toxics13010013/s1, Figure S1. Plastitar formations that were found at different sites located along the Ionian coast of the Apulian Region; Figure S2: 1H- NMR spectrum of the plastitar compound in CD_2_Cl_2_. Integrated hydrogen signals are indicated; Figure S3: Some pellets were melted together when extracted from tar; this is from sample P8. The pellet was not divided during the analysis and was considered a single MP. Scale in the picture, 0.5 mm; Figure S4: Brown pellets found within the sample P4 with visible weathering and encrustation due to the exposure to environmental factors. Scale in the picture, 0.5 mm; Figure S5: ^1^H-^13^C hsqc spectrum of the plastitar compound in CDCl_3_ solvent. Carbon assignments are indicated.

## References

[1] Villarrubia-Gómez P., Cornell S.E., Fabres J. (2018). Marine Plastic Pollution as a Planetary Boundary Threat—The Drifting Piece in the Sustainability Puzzle. Mar. Policy.

[2] MacLeod M., Arp H.P.H., Tekman M.B., Jahnke A. (2021). The Global Threat from Plastic Pollution. Science.

[3] Pinheiro L.M., Ivar Do Sul J.A., Costa M.F. (2020). Uptake and Ingestion Are the Main Pathways for Microplastics to Enter Marine Benthos: A Review. Food Webs.

[4] (2023). Plastics—The Fast Facts 2023.

[5] Think That Your Plastic Is Being Recycled?. Think Again..

[6] Miller M.E., Hamann M., Kroon F.J. (2020). Bioaccumulation and Biomagnification of Microplastics in Marine Organisms: A Review and Meta-Analysis of Current Data. PLoS ONE.

[7] Chen G., Li Y., Wang J. (2021). Occurrence and Ecological Impact of Microplastics in Aquaculture Ecosystems. Chemosphere.

[8] Arijeniwa V.F., Akinsemolu A.A., Chukwugozie D.C., Onawo U.G., Ochulor C.E., Nwauzoma U.M., Kawino D.A., Onyeaka H. (2024). Closing the Loop: A Framework for Tackling Single-Use Plastic Waste in the Food and Beverage Industry through Circular Economy—A Review. J. Environ. Manag..

[9] Cózar A., Echevarría F., González-Gordillo J.I., Irigoien X., Úbeda B., Hernández-León S., Palma Á.T., Navarro S., García-de-Lomas J., Ruiz A. (2014). Plastic Debris in the Open Ocean. Proc. Natl. Acad. Sci. USA.

[10] Jambeck J.R., Geyer R., Wilcox C., Siegler T.R., Perryman M., Andrady A., Narayan R., Law K.L. (2015). Plastic Waste Inputs from Land into the Ocean. Science.

[11] Suaria G., Avio C.G., Mineo A., Lattin G.L., Magaldi M.G., Belmonte G., Moore C.J., Regoli F., Aliani S. (2016). The Mediterranean Plastic Soup: Synthetic Polymers in Mediterranean Surface Waters. Sci. Rep..

[12] Haward M. (2018). Plastic Pollution of the World’s Seas and Oceans as a Contemporary Challenge in Ocean Governance. Nat. Commun..

[13] Cressey D. (2016). The plastic ocean. Nature..

[14] Jeyasanta K.I., Sathish N., Patterson J., Edward J.K.P. (2020). Macro-, Meso- and Microplastic Debris in the Beaches of Tuticorin District, Southeast Coast of India. Mar. Pollut. Bull..

[15] Haarr M.L., Falk-Andersson J., Fabres J. (2022). Global Marine Litter Research 2015–2020: Geographical and Methodological Trends. Sci. Total Environ..

[16] Andrady A.L. (2011). Microplastics in the Marine Environment. Mar. Pollut. Bull..

[17] De Benedetto G.E., Fraissinet S., Tardio N., Rossi S., Malitesta C. (2024). Microplastics Determination and Quantification in Two Benthic Filter Feeders Sabella Spallanzanii, Polychaeta and Paraleucilla Magna, Porifera. Heliyon.

[18] Materić D., Holzinger R., Niemann H. (2022). Nanoplastics and Ultrafine Microplastic in the Dutch Wadden Sea—The Hidden Plastics Debris?. Sci. Total Environ..

[19] Fraissinet S., De Benedetto G.E., Malitesta C., Holzinger R., Materić D. (2024). Microplastics and Nanoplastics Size Distribution in Farmed Mussel Tissues. Commun. Earth Environ..

[20] Gong Y., Zhao X., Cai Z., O’Reilly S.E., Hao X., Zhao D. (2014). A Review of Oil, Dispersed Oil and Sediment Interactions in the Aquatic Environment: Influence on the Fate, Transport and Remediation of Oil Spills. Mar. Pollut. Bull..

[21] Frasier K.E., Solsona-Berga A., Stokes L., Hildebrand J.A., Murawski S.A., Ainsworth C.H., Gilbert S., Hollander D.J., Paris C.B., Schlüter M., Wetzel D.L. (2020). Impacts of the Deepwater Horizon Oil Spill on Marine Mammals and Sea Turtles. Deep Oil Spills.

[22] Duarte C.M., Agusti S., Barbier E., Britten G.L., Castilla J.C., Gattuso J.-P., Fulweiler R.W., Hughes T.P., Knowlton N., Lovelock C.E. (2020). Rebuilding Marine Life. Nature.

[23] Kalter V., Passow U. (2023). Quantitative Review Summarizing the Effects of Oil Pollution on Subarctic and Arctic Marine Invertebrates. Environ. Pollut..

[24] Asif Z., Chen Z., An C., Dong J. (2022). Environmental Impacts and Challenges Associated with Oil Spills on Shorelines. J. Mar. Sci. Eng..

[25] Huettel M. (2022). Oil Pollution of Beaches. Curr. Opin. Chem. Eng..

[26] D’Affonseca F.M., Vieira Reis F.A.G., Corrêa C.V.D.S., Wieczorek A., Giordano L.D.C., Marques M.L., Rodrigues F.H., Costa D.M., Kolya A.D.A., Veiga V.M. (2023). Environmental Sensitivity Index Maps to Manage Oil Spill Risks: A Review and Perspectives. Ocean Coast. Manag..

[27] Gundlach E.R., Hayes M.O. (1978). Vulnerability of Coastal Environments to Oil Spill Impacts. Mar. Technol. Soc. J..

[28] Domínguez-Hernández C., Villanova-Solano C., Sevillano-González M., Hernández-Sánchez C., González-Sálamo J., Ortega-Zamora C., Díaz-Peña F.J., Hernández-Borges J. (2022). Plastitar: A New Threat for Coastal Environments. Sci. Total Environ..

[29] Saliu F., Lasagni M., Clemenza M., Chubarenko I., Esiukova E., Suaria G. (2023). The Interactions of Plastic with Tar and Other Petroleum Derivatives in the Marine Environment: A General Perspective. Mar. Pollut. Bull..

[30] Ellrich J.A., Ehlers S.M., Furukuma S. (2023). Plastitar Records in Marine Coastal Environments Worldwide from 1973 to 2023. Front. Mar. Sci..

[31] Turner A., Holmes L. (2011). Occurrence, Distribution and Characteristics of Beached Plastic Production Pellets on the Island of Malta (Central Mediterranean). Mar. Pollut. Bull..

[32] Fajković H., Cuculić V., Cukrov N., Kwokal Ž., Pikelj K., Huljek L., Marinović S. (2020). Plasto-Tarball—A Sinkhole for Microplastic (Croatian Coast Case Study). MICRO.

[33] Saliu F., Compa M., Becchi A., Lasagni M., Collina E., Liconti A., Suma E., Deudero S., Grech D., Suaria G. (2023). Plastitar in the Mediterranean Sea: New Records and the First Geochemical Characterization of These Novel Formations. Mar. Pollut. Bull..

[34] Sharma S., Sharma V., Chatterjee S. (2021). Microplastics in the Mediterranean Sea: Sources, Pollution Intensity, Sea Health, and Regulatory Policies. Front. Mar. Sci..

[35] MARPOL 1973—Final Act and Convention.Pdf, n.d.—Cerca Con Google. https://wwwcdn.imo.org/localresources/en/KnowledgeCentre/ConferencesMeetings/Documents/MARPOL%201973%20-%20Final%20Act%20and%20Convention.pdf.

[36] Carlucci R., Capezzuto F., Cipriano G., D’Onghia G., Fanizza C., Libralato S., Maglietta R., Maiorano P., Sion L., Tursi A. (2021). Assessment of Cetacean–Fishery Interactions in the Marine Food Web of the Gulf of Taranto (Northern Ionian Sea, Central Mediterranean Sea). Rev. Fish Biol. Fish..

[37] Schneider C.A., Rasband W.S., Eliceiri K.W. (2012). NIH Image to ImageJ: 25 Years of Image Analysis. Nat. Methods.

[38] Poveda J.C., Molina D.R. (2012). Average Molecular Parameters of Heavy Crude Oils and Their Fractions Using NMR Spectroscopy. J. Pet. Sci. Eng..

[39] Oliviero Rossi C., Caputo P., De Luca G., Maiuolo L., Eskandarsefat S., Sangiorgi C. (2018). 1H-NMR Spectroscopy: A Possible Approach to Advanced Bitumen Characterization for Industrial and Paving Applications. Appl. Sci..

[40] Nciri N., Song S., Kim N., Cho N. (2014). Chemical Characterization of Gilsonite Bitumen. J. Pet. Environ. Biotechnol..

[41] Nciri N., Kim N., Cho N. (2017). New Insights into the Effects of Styrene-Butadiene-Styrene Polymer Modifier on the Structure, Properties, and Performance of Asphalt Binder: The Case of AP-5 Asphalt and Solvent Deasphalting Pitch. Mater. Chem. Phys..

[42] Poveda J.C., Molina And D.-R., Pantoja-Agreda E.-F. (2014). 1H- and 13C-NMR Structural Characterization of Asphaltenes from Vacuum Residua Modified by Thermal Cracking. CTF-Cienc. Tecnol. Futuro.

[43] Majid A., Pihillagawa I. (2014). Potential of NMR Spectroscopy in the Characterization of Nonconventional Oils. J. Fuels.

[44] Read J., Whiteoak D. (2003). The Shell Bitumen Handbook.

[45] Porto M., Angelico R., Caputo P., Abe A.A., Teltayev B., Rossi C.O. (2022). The structure of bitumen: Conceptual models and experimental evidences. Materials.

[46] Galgani F., Rangel-Buitrago N. (2024). White Tides: The Plastic Nurdles Problem. J. Hazard. Mater..

[47] Erni-Cassola G., Zadjelovic V., Gibson M.I., Christie-Oleza J.A. (2019). Distribution of Plastic Polymer Types in the Marine Environment; A Meta-Analysis. J. Hazard. Mater..

[48] Primpke S., Fischer M., Lorenz C., Gerdts G., Scholz-Böttcher B.M. (2020). Comparison of Pyrolysis Gas Chromatography/Mass Spectrometry and Hyperspectral FTIR Imaging Spectroscopy for the Analysis of Microplastics. Anal. Bioanal. Chem..

[49] Campanale C., Savino I., Massarelli C., Uricchio V.F. (2023). Fourier Transform Infrared Spectroscopy to Assess the Degree of Alteration of Artificially Aged and Environmentally Weathered Microplastics. Polymers.

[50] Niaounakis M., Kontou E., Pispas S., Kafetzi M., Giaouzi D. (2019). Aging of Packaging Films in the Marine Environment. Polym. Eng. Sci..

[51] Di Giulio T., De Benedetto G.E., Ditaranto N., Malitesta C., Mazzotta E. (2024). Insights into Plastic Degradation Processes in Marine Environment by X-Ray Photoelectron Spectroscopy Study. Int. J. Mol. Sci..

[52] Marchetto D., De Ferri L., Latella A., Pojana G. (2022). Micro- and Mesoplastics in Sea Surface Water from a Northern Adriatic Coastal Area. Environ. Sci. Pollut. Res..

[53] Renner G., Sauerbier P., Schmidt T.C., Schram J. (2019). Robust Automatic Identification of Microplastics in Environmental Samples Using FTIR Microscopy. Anal. Chem..

[54] Andrady A.L. (2022). Weathering and Fragmentation of Plastic Debris in the Ocean Environment. Mar. Pollut. Bull..

[55] Roy Chowdhury P., Medhi H., Bhattacharyya K.G., Hussain C.M. (2023). Emerging Plastic Litter Variants: A Perspective on the Latest Global Developments. Sci. Total Environ..

[56] Prokić M.D., Gavrilović B.R., Radovanović T.B., Gavrić J.P., Petrović T.G., Despotović S.G., Faggio C. (2021). Studying Microplastics: Lessons from Evaluated Literature on Animal Model Organisms and Experimental Approaches. J. Hazard. Mater..

[57] Prokić M.D., Radovanović T.B., Gavrić J.P., Faggio C. (2019). Ecotoxicological Effects of Microplastics: Examination of Biomarkers, Current State and Future Perspectives. TrAC Trends Anal. Chem..

