# Supplementary material for: New Plastitar Record for the Mediterranean Sea: Characterization of Plastics and Tar from the Salento Peninsula (Ionian Sea)
Source: Toxics. 2024 Dec 26;13(1):13. doi: 10.3390/toxics13010013 (PMC11768737; doi:10.3390/toxics13010013)
Supplement: Supplementary file 1 [file toxics-13-00013-s001.zip › toxics-3373972-supplementary.pdf]

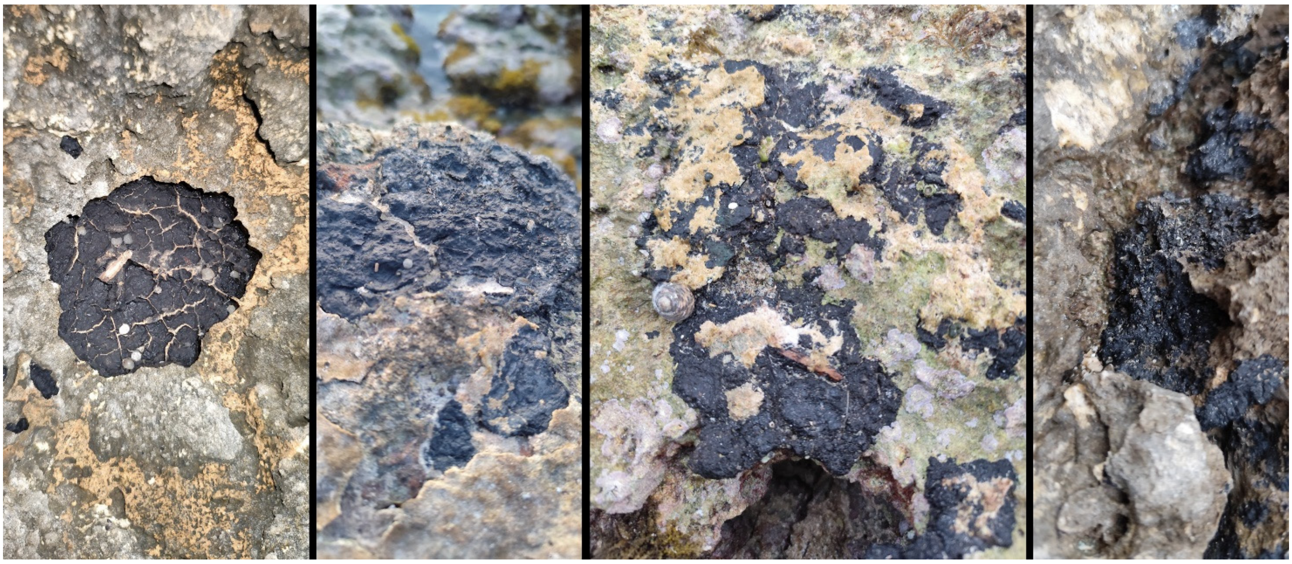

Figure S1. Plastitar formations that were found in different sites located along the Ionian coast of the Apulian Region.

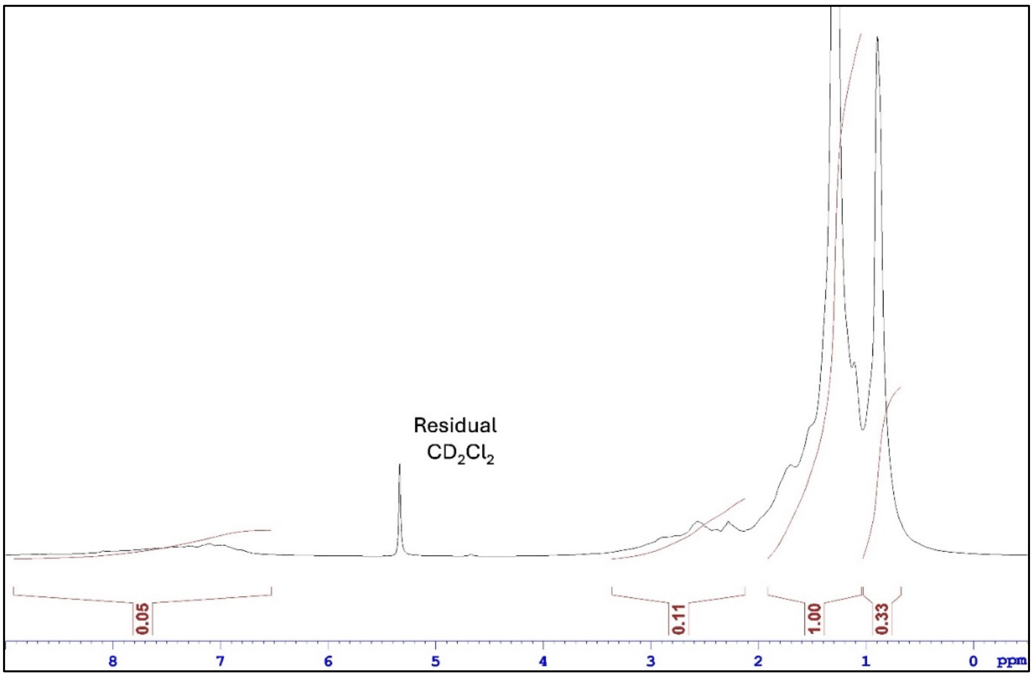

Figure S2. ^{1}H- NMR spectrum of Plastitar compound in CD_2Cl_2. Integrated hydrogen signals are indicated.

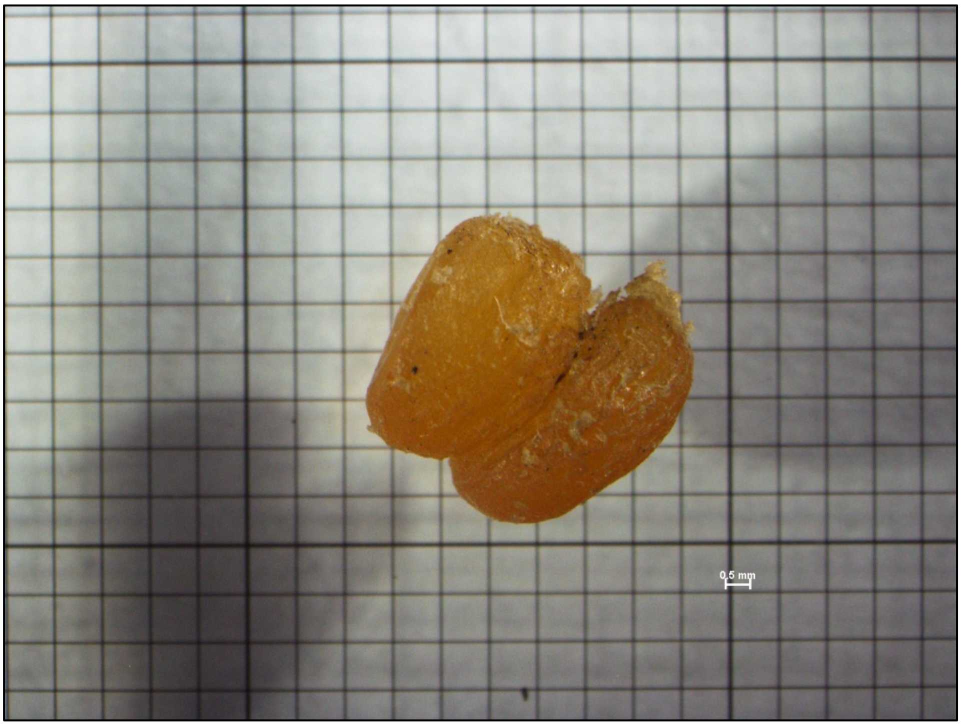

Figure S3. Some pellets were melted together when extracted from tar, this is from sample P8. The pellet weren't divided during the analysis and were considered as a single MPs. Scale in the picture 0.5 mm

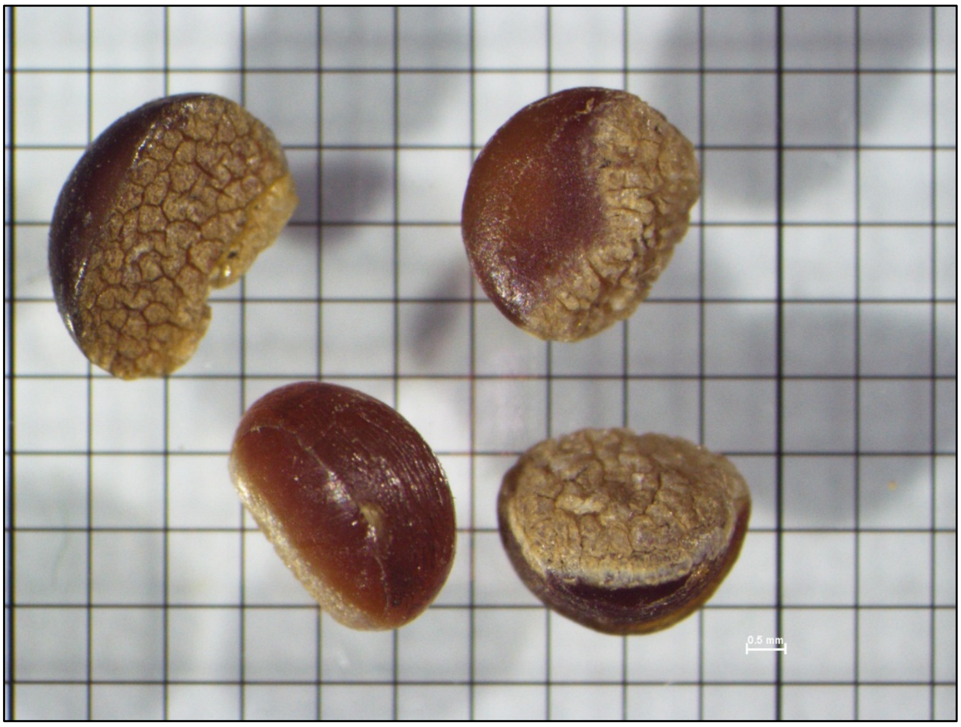

Figure S4. Brown pellets found within the sample P4 with visible weathering and encrustation due to the exposure to environmental factors. Scale in the picture 0.5 mm.

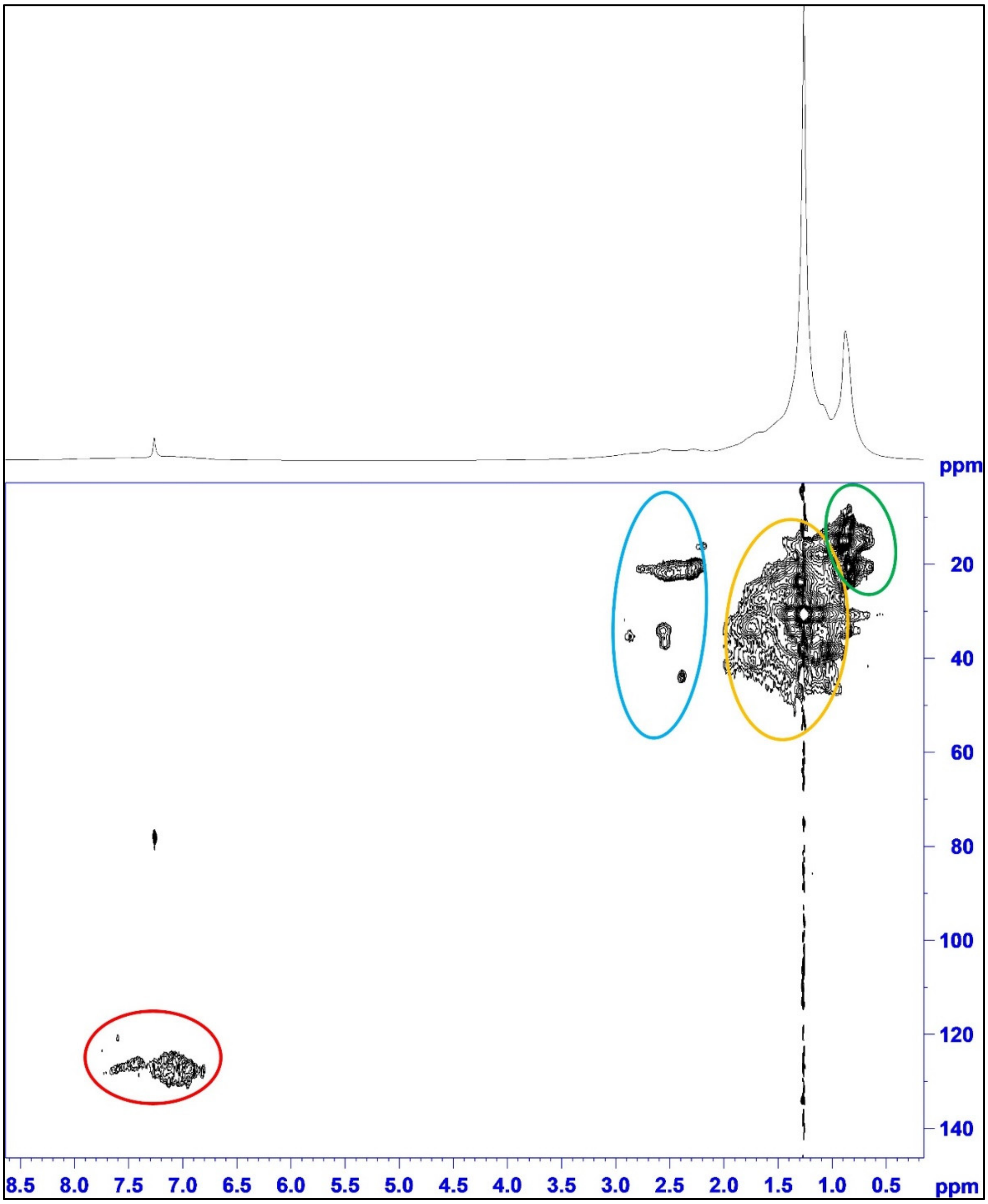

Figure S5. ^{1}H-^{13}C hsqc spectrum of Plastitar compound in $CDCl_3$ solvent. Carbon assignments are indicated.
